# Assessing Prescribing Patterns of Nonstatins as Add-on Therapy for Secondary Prevention in a Federally Qualified Health Center

**DOI:** 10.3390/pharmacy13050129

**Published:** 2025-09-06

**Authors:** Sabrina Guerra, Kathryn P. Lin, Ahmed S. Kenawy, Chanhyun Park, Morgan P. Stewart

**Affiliations:** 1 College of Pharmacy, The University of Texas at Austin, Austin, TX 78712, USA; 2 Communitycare Health Centers, Austin, TX 78723, USA

**Keywords:** cardiovascular diseases, dyslipidemia, anticholesteremic agents, LDL cholesterol, clinical practice patterns, secondary prevention

## Abstract

Background: Lipid management is a key aspect of secondary atherosclerotic cardiovascular disease (ASCVD) prevention. However, real-world studies show that ~72–88% of patients with ASCVD fail to meet their low-density lipoprotein cholesterol (LDL-C) target. Nonstatin agents are available as add-on therapies that can be utilized when maximally tolerated statins are insufficient to achieve LDL-C goals. This retrospective study aimed to evaluate the current prescribing habits of nonstatins as add-on therapy to statins for secondary ASCVD prevention at a federally qualified health center (FQHC). Methods: Patients were included if they had a history of clinical ASCVD, ≥1 lipid panel obtained during the study period, and were prescribed any intensity statin. Results: Among 398 included participants, 11.1% were prescribed nonstatin therapy and 35.9% were meeting a LDL-C target of <70 mg/dL. There was a significant association between being prescribed ezetimibe based on the type of healthcare coverage (*p* = 0.04) and a higher number of ASCVD qualifying indications (*p* < 0.01). Conclusions: Overall, nonstatins were found to be underutilized for LDL-C management in this underserved population. Future initiatives should target ways to optimize nonstatin therapy to optimize secondary ASCVD prevention.

## 1. Introduction

Cardiovascular diseases (CVD) are the most common cause of death in the United States and globally [1,2,3]. Nearly 18 million global deaths per year can be attributed to cardiovascular disease, such as coronary heart disease and stroke [4]. A major indicator of increased risk of cardiovascular diseases, particularly atherosclerotic cardiovascular disease (ASCVD), is dyslipidemia. Lipid management is a key aspect of secondary atherosclerotic cardiovascular disease (ASCVD) prevention. Current guidelines recommend a low-density lipoprotein (LDL) goal of at least <70 mg/dL for many patients with a history of ASCVD [5]. Despite the known mortality and morbidity benefits of optimally treating dyslipidemia in patients with ASCVD, the data reveals that dyslipidemia remains undertreated. Real-world studies show that ~72–88% of patients with ASCVD fail to meet this target [6,7,8,9].

While HMG-CoA reductase inhibitors, commonly known as “statins”, are the first-line treatment option for dyslipidemia, within the past 20 years, there has been a rise in nonstatin medications available to treat patients who are not at goal with maximally tolerated statin use or who need a statin alternative. Three nonstatin medications with cardiovascular mortality benefit in patients with a history of ASCVD are currently highlighted as second-line options by the American College of Cardiology: ezetimibe, which reduces the absorption of cholesterol, and alirocumab and evolocumab, which are proprotein convertase subtilisin/kexin type 9 inhibitors (PCSK9i) [5,10,11,12]. Ezetimibe is an oral, often low-cost agent that can lower LDL-C ~18% when added to statin therapy [10]. PCSK9i are refrigerated injectables administered subcutaneously that lower LDL-C ~60% when added to statins [11,12]. However, they are often more expensive or require prior authorizations for patients with insurance [13]. Other third and fourth line nonstatin agents exist, including bempedoic acid and inclisiran [5].

Unfortunately, disparities in prescribing statin therapies have been documented, particularly for female, black, and Hispanic patients [14,15,16]. Federally qualified health centers (FQHCs) serve as critical healthcare providers for underserved populations. Social drivers (or determinants) of health (SDOH) are nonmedical factors, such as access to transportation, that can be a large factor in potential outcomes of chronic disease management [17]. Managing dyslipidemia and ASCVD prevention for underserved populations presents challenges such as financial constraints limiting affordability of medications, particularly beyond statins; access to healthy food options to adhere to lipid-lowering lifestyle recommendations; and low education regarding risks, among other SDOH factors. Therefore, patients may experience suboptimal lipid control, leading to higher rates of cardiovascular events. Reducing gaps in prescribing and access to medications is one way to consciously care for patients at risk. One opportunity to improve is by optimizing the use of pharmacists, who are uniquely positioned to aid with access concerns, adherence, and improving population health metrics, including statin prescribing [18]. It is unknown if there are disparities in nonstatin prescribing among FQHC patients.

This study aimed to evaluate the current prescribing habits of nonstatin agents as add-on therapy to statins for secondary ASCVD prevention at a FQHC system in Travis County, Texas, and to compare characteristics of patients who had reached an LDL-C goal of <70 mg/dL with those who did not.

## 2. Materials and Methods

### 2.1. Data Source

We conducted this study using the electronic health record (EHR) of CommUnityCare Health Centers in Austin, Texas. This EHR is powered by the EPIC system, which provides a broad range of clinical, financial, and administrative variables. These variables include patient demographics (e.g., sex, race, insurance status, income level, and employment status), clinical data (e.g., diagnoses, allergies, medications, immunizations, and lab results), Social Determinants of Health (e.g., food insecurity, housing, transportation, education, and employment), claims data, and visit data (e.g., date, type, reason, services, and progress notes).

### 2.2. Study Design and Study Population

**Figure 1:**
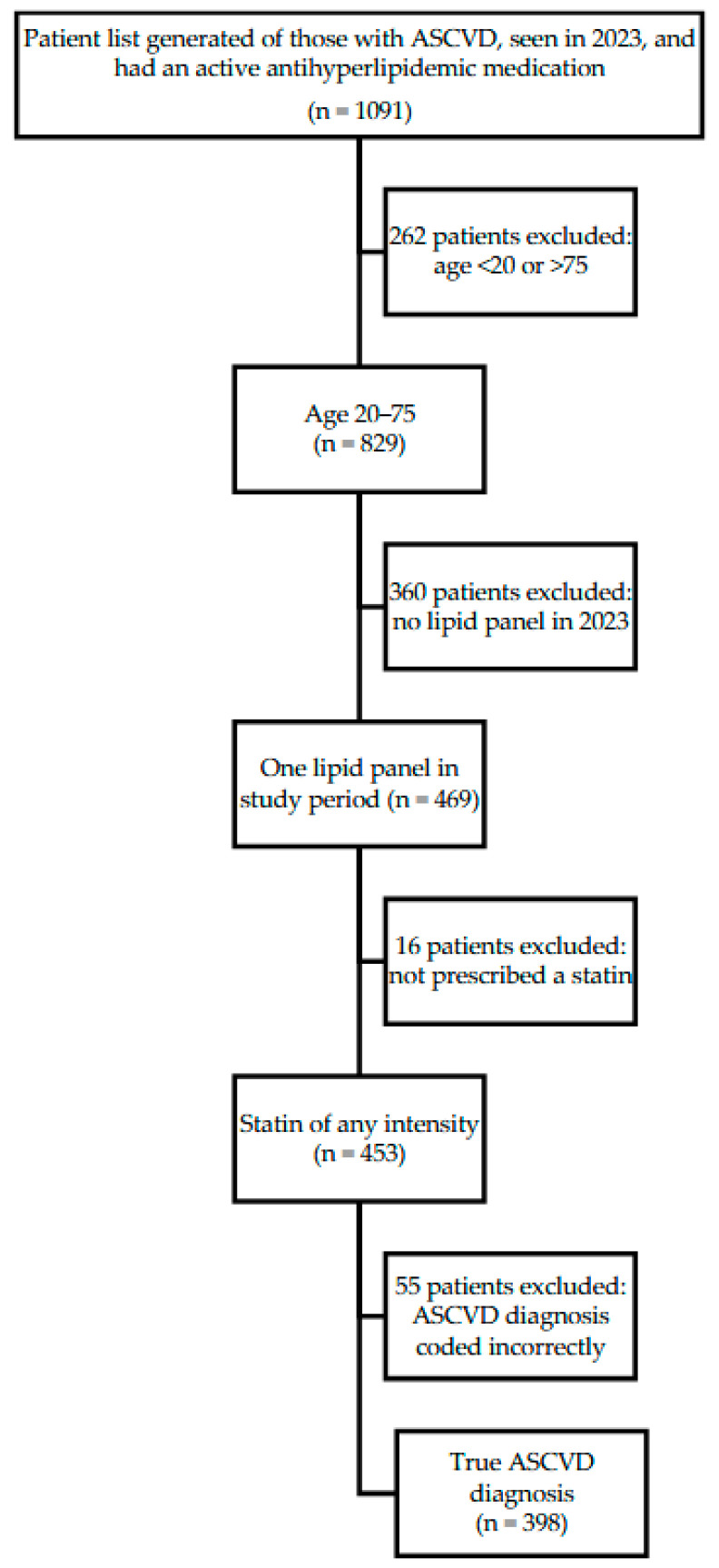
Study Population Inclusion Criteria.

This was a retrospective observational study of patients of a FQHC system in Travis, County, TX. A report from the EHR was generated of patients who (1) had been seen by a provider between January 1 and Dec 31, 2023, (2) had a documented history of clinical ASCVD, and (3) were prescribed any antihyperlipidemic medication (Figure 1).

Individuals were screened for inclusion if they obtained at least one lipid panel during the study period and were prescribed a statin of any intensity. Individuals were excluded if they were outside the age range of 20 to 75 years, or if upon chart review the qualifying ASCVD diagnosis was coded incorrectly (e.g., a hemorrhagic stroke incorrectly coded as an ischemic stroke).

In alignment with clinical practice guidelines, a history of clinical ASCVD was defined as any history of acute coronary syndrome, history of myocardial infarction, stable or unstable angina, coronary or other arterial revascularization, ischemic stroke, transient ischemic attack or peripheral artery disease presumed to be of atherosclerotic origin [5]. Demographic information, body mass index (BMI), ASCVD qualifying diagnoses, LDL-C values, SDOH factors, lipid-lowering medications, and attendance at clinical pharmacist appointments were collected. SDOH factors within this FQHC system are patient-reported responses to an annual questionnaire that screens for potential non-medical drivers of care including: tobacco use, alcohol use, financial resource strain, transportation needs, stress, intimate partner violence, housing stability, food insecurity, physical activity, social connections, depression, and health literacy. Each section of this screening tool has a pre-defined cutoff based on patient response for low, medium, or high risk.

This health system includes over 30 clinic sites across the Austin region and delivers both primary and specialty care to more than 130,000 individuals, most of whom lack adequate health insurance coverage. Patients commonly access healthcare services and prescription medications through a county-supported Medical Access Program (MAP) or a locally administered sliding fee scale (SFS). Of note, the system employs a clinical pharmacy team consisting of 17 pharmacists throughout the system. Clinical pharmacists at this FQHC provide comprehensive medication management under a collaborative practice agreement with physicians, which includes being able to order labs as well as initiate and adjust dyslipidemia therapies as clinically indicated and under protocol.

This study was deemed exempt by the University of Texas at Austin Institutional Review Board (STUDY00003973).

### 2.3. Outcome

The primary outcome was the proportion of patients using nonstatin agents as add-on therapy to statins for secondary ASCVD prevention. Secondary outcomes were to describe associations between ezetimibe use and patient characteristics and to describe differences between patients who met vs. did not meet the LDL-C goal. A uniform LDL-C goal was defined as <70 mg/dL, for all patients to be consistent with expert guidance recommending this threshold, to consider the addition of nonstatin therapy to maximally tolerated statins in patients with ASCVD [5].

### 2.4. Statistical Analysis

Descriptive statistics were used to summarize the study population. Patients were divided into ezetimibe users and non-users, as well as those with LDL-C < 70 mg/dL and ≥70 mg/dL. Chi-square or Fisher’s exact tests were used to compare categorical variables between these groups. The Wilcoxon rank-sum test was used for comparing continuous variables. A *p*-value < 0.05 was considered statistically significant. The statistical analysis was conducted using R for Statistics version 4.4.1 [19].

## 3. Results

An initial report found 1091 patients. Of these, 398 met the full inclusion criteria (See Figure 1). Demographics are included in Table 1. The majority of included individuals were White (65.30%) and male (61.10%), with an average age of 59.27 years (SD = 8.50), and a mean BMI of 31.51 (SD = 7.30). Most patients (51.5%) had no primary health insurance coverage but were instead receiving care through a local health coverage program (MAP) for low-income residents. Most patients had one ASCVD-qualifying diagnosis (53.50%), were prescribed a high-intensity statin (77.10%), and did not see a clinical pharmacist during the study period (74.87%). Among the 398 study participants, 9.5% (38/398) were prescribed ezetimibe, and 35.9% were meeting an LDL-C target of <70 mg/dL. Only six participants were prescribed a PCSK9i, all of which were for evolocumab; only one patient was prescribed bempedoic acid as a combination tablet with ezetimibe and was included in the ezetimibe group. Due to the low number of participants in non-ezetimibe groups, only those prescribed ezetimibe were compared for subgroup statistical analyses. All of the participants that were prescribed a nonstatin were on a high-intensity statin except for eight. All six patients on ezetimibe were on their maximum tolerated statin due to allergy or intolerance, and it was not documented why the two patients prescribed a PCSK9i were not on a high-intensity statin. There was a significant association between being prescribed ezetimibe based on the type of healthcare coverage (*p* = 0.04), and a higher number of ASCVD-qualifying indications (*p* < 0.01) (Table 2). Of the 38 participants on ezetimibe, 10 (26.3%) were meeting the LDL goal < 70 mg/dL. Of those, one was on a moderate-intensity statin and nine were on a high-intensity statin. Of the 28 participants who were not at LDL goal, five were on a moderate- or low-intensity statin and 23 were on a high-intensity statin. This association was not significant with a *p*-value of 0.56. In addition, there was a significant relationship between meeting an LDL-C goal of <70 and the participant’s ethnicity and lower BMI (*p* = 0.01, for both) (Table 3). While not statistically significant, there was a trend towards significance for the relationship between receiving a prescription for ezetimibe and having an appointment with a clinical pharmacist (*p* = 0.05).

## 4. Discussion

The results of this study show that while the percentage of patients with clinical ASCVD meeting their LDL-C targets is higher than in other real-world studies (35.9% vs. <30%), there is still a significant amount of work to be undertaken in optimizing lipid-lowering therapies [6,7,8,9]. Furthermore, this study confirms that nonstatin use remains underutilized in underserved settings, which has significant implications for the healthcare system. Both findings allude to downstream negative consequences, which include higher risks of recurrent ASCVD in groups that are not meeting their LDL-C targets and the associated healthcare and quality of life costs of disease burden. Clinical inertia, medication adherence, and access to therapy have been suggested as the most common reasons patients with clinical ASCVD do not achieve LDL-C targets; all of these likely played a role in this healthcare system [6].

All patients prescribed a nonstatin were on their maximally tolerated statin per guideline recommendations (except two with limited documentation). In this study, the only patient-specific factors significantly associated with receiving a prescription of ezetimibe as add-on therapy to a statin were the type of healthcare coverage and the number of qualifying ASCVD indications. A greater proportion of patients with MAP coverage were prescribed ezetimibe compared to those with other types of insurance. This may reflect prescribers’ greater familiarity with MAP formulary options in this FQHC system, rather than representing a true patient-specific driver of lipid-lowering therapy intensification. Conversely, patients with two or more ASCVD-qualifying conditions were also more likely to receive ezetimibe, suggesting that clinicians may be more proactive in escalating lipid-lowering therapy in patients with a history of recurrent ASCVD events or the presence of multiple ASCVD conditions.

This study also compared patients who were or were not meeting their LDL-C goal to identify disparities and opportunities to overcome barriers for prescribing nonstatins. A significant association was observed between meeting an LDL-C goal of <70 mg/dL and both a lower BMI and Hispanic/Latino ethnicity. Clinically, this makes sense as lowering weight lowers LDL-C. However, this statistical finding may be of limited clinical significance as both mean BMI values (30 and 32) fall within the category of Class One obesity. As seen in previous studies, ethnic disparities in achieving LDL-C goals were observed in this cohort, though to a lesser degree, given the high prevalence of Hispanic/Latino individuals receiving MAP coverage, which was associated with a higher chance of being prescribed ezetimibe. Targeted interventions for Hispanic and other minority individuals may still be needed in other settings to ensure health equity in underserved populations.

In addition to disparities in reaching LDL-C goals, medication-specific factors also influenced the underutilization of nonstatin agents, particularly PCSK9i medications, which had a very low utilization in this analysis (1.5%). Upon a post hoc chart review of clinical notes, only two of the six individuals appeared to be able to access and use the medication, with the other four citing cost issues that were prohibiting access. This finding is consistent with other analyses indicating similar trends [6,21]. Several reasons may have contributed to higher levels of ezetimibe being prescribed over PCSK9is and other nonstatins in this study. Ezetimibe is a generic medication and easily accessible for many patients. PCSK9i medications often require a prior authorization or are more expensive [13,22]. For patients without insurance, prescription patient assistance programs (PAP) are available through the manufacturers. Though medications obtained through PAP would eventually be provided at no cost to the patient, they require many additional administrative steps and care coordination that providers and patients may be unaware of or have other barriers to completing. Additionally, the recommendations, including PCSK9i as second line agents rather than third line considerations after adding on ezetimibe due to cost, had recently changed in September 2022 [5]. Clinician practice may have lagged in adapting this recommendation, leading to less frequent prescribing of PCSK9i. Bempedoic acid is recommended as a third line agent. Though it is an oral agent, it is not often covered by insurance, it is not on the MAP formulary, and there is no PAP for it. The chart review revealed that the patient who was prescribed the bempedoic acid-ezetimibe combination tablet was not able to access it and was switched to ezetimibe alone.

Multiple studies have described the positive impact of pharmacist-led interventions on lipid management, particularly in improving statin use across diverse clinical settings [18]. In this study, although the association between having a clinical pharmacist appointment and being prescribed ezetimibe did not reach statistical significance (*p* = 0.05), it did suggest that individuals engaging with a clinical pharmacist were more likely to be receiving ezetimibe as add-on therapy to statins. This finding may not have reached statistical significance due to the relatively low number of individuals prescribed ezetimibe overall, limiting the power to detect a meaningful difference. While the study design did not allow for confirmation of whether the clinical pharmacist directly initiated the ezetimibe prescription, this study reinforces the potential role of pharmacists in addressing care gaps and optimizing nonstatin therapies for patients with clinical ASCVD. In contrast, having an appointment with a clinical pharmacist was not associated with achieving an LDL-C goal of <70 mg/dL; however, this may reflect the complex nature of patients referred to pharmacists and highlight continued opportunities for pharmacists, alongside physicians and other advanced providers, to lead targeted education and therapy optimization interventions for patients who are at high risk for ASCVD. Examples may include ensuring screening and lipid panels are up to date, patient education handouts or classes about ASCVD, LDL-C goals, and nonstatins, or dedicated appointments regarding dyslipidemia. Pharmacists, specifically, can aid with education to providers regarding access to nonstatins to increase prescribing, particularly of PCSK9-i when best indicated. Future research should explore the impact of pharmacist-led interventions related to nonstatin agents to better understand and leverage their contribution to improving cardiovascular outcomes, especially in underserved settings.

There are some limitations of this study, which include this study being a snapshot of 2023 for these patients. The low number of patients on a nonstatin in our sample may have limited our ability to detect meaningful effects of their use. Our analysis did not demonstrate a significant difference in LDL-C goal attainment among patients prescribed ezetimibe, despite prior studies indicating improved LDL-C [10]. Some patients may have started seeing a clinical pharmacist or taking medications after the study period. One notable limitation of this study is the use of a uniform LDL-C goal of <70 mg/dL for all patients with ASCVD, despite expert guidance that patients at ‘very high risk’ for recurrent events should aim for an LDL-C goal of <55 mg/dL [5]. Due to limitations in the EHR reporting capabilities, we were unable to reliably identify which patients met clinical criteria for the more stringent LDL-C goal. This may have led to an overestimation of goal attainment and limited our ability to differentiate between high-risk and very high-risk patients within the ASCVD population.

Furthermore, one important limitation of this study is the inclusion and exclusion criteria, which may have inadvertently introduced selection bias by capturing a more engaged subset of the population. Eligibility for the study required that patients had been seen within the past year, have an active statin prescription, and have completed a lipid panel during the study period, criteria that may exclude individuals who are less connected to the healthcare system. Notably, patients with clinical ASCVD who had not undergone recent lipid testing or had never been initiated on statin therapy were not represented in this analysis. This limits the generalizability of the findings and underrepresents a potentially high-risk group that may have greater unmet needs. Lipid profile assessment is a cornerstone of secondary ASCVD prevention and should occur at least annually, or more frequently when therapy is adjusted. The absence of timely lipid monitoring, as evidenced by the large number of excluded individuals, may reflect clinical inertia, competing priorities during visits, system-level barriers, or challenges related to patient access. Addressing these gaps in routine lipid testing and statin initiation represents a critical opportunity to improve cardiovascular care and ensure timely intensification of therapy for all patients with ASCVD.

Future research initiatives should target ways to ensure routine monitoring, intensify lipid-lowering therapy where appropriate, and optimize secondary ASCVD prevention in the system. Potential interventions in future studies include healthcare provider education, targeted population health outreach, and utilizing clinical pharmacists for medication intervention.

## 5. Conclusions

Overall, nonstatins were found to be underutilized for LDL-C management in this underserved population, despite not meeting LDL-C goals. Likewise, ezetimibe is a low-cost medication that is underutilized for LDL-C management in this population. Future initiatives should target ways to intensify lipid-lowering therapy and optimize secondary ASCVD prevention in the system. Potential interventions could include healthcare provider education, targeted population health outreach, and utilizing clinical pharmacists for medication intervention.

## Figures and Tables

**Table 1:** Patient Characteristics (*n* = 398).

Variable	Mean ± SD (Median)
Age	59.27 ± 8.50 (60)
BMI	31.51 ± 7.30 (30.23)
	**N (%)**
Sex	
Male	243 (61.06)
Female	155 (38.94)
Race	
Unreported	33 (8.29)
American Indian/Alaska Native	2 (0.50)
Asian	18 (4.52)
Black/African American	76 (19.10)
More than one race	3 (0.75)
Other	6 (1.51)
White	260 (65.32)
Ethnicity	
Non-Hispanic/Non-Latino	183 (45.98)
Hispanic/Latino	187 (46.98)
Unreported/Refused	28 (7.04)
Primary Coverage	
MAP	205 (51.51)
Medicaid	50 (12.56)
Medicare	100 (25.13)
Private	32 (8.04)
Self-Pay	10 (2.51)
Tricare	1 (0.25)
Statin Intensity Category **	
Low	7 (1.76)
Moderate	84 (21.11)
High	307 (77.14)
High-Risk SDOH	
0	154 (38.69)
1–2	127 (31.91)
3–5	88 (22.11)
6+	29 (7.29)
Number of ASCVD Indications	
1	212 (53.27)
2+	186 (46.73)
LDL-C < 70 mg/dL	
Yes	143 (35.93)
No	255 (64.07)
Ezetimibe Use	
Yes	38 (9.55) *
No	360 (90.45)
PCSK9i Use	
Yes	6 (1.51)
No	392 (98.49)
Other Nonstatin	
Yes *	1 (0.25)
No	397 (99.75)
Clinical Pharmacist Visit in 2023	
No	298 (74.87)
Yes	100 (25.13)

* 1 patient was prescribed the bempedoic acid-ezetimibe combination tablet. This patient was counted in the other nonstatin group and the ezetimibe group. ** Statin intensity is determined by LDL-C lowering. Low intensity: <30%, Moderate intensity: 30–50%, High intensity: >50% [20].

**Table 2:** Variables by Ezetimibe Use.

Variable	Use of Ezetimibe (*n* = 38)	No Use of Ezetimibe (*n* = 360)	*p*-Value
	**Mean ± SD (Median)**	**Mean ± SD (Median)**	
Age ^1^	59.70 ± 6.65 (59.00)	59.20 ± 8.68 (60.50)	0.98
BMI ^1^	32.90 ± 7.12 (31.10)	31.40 ± 7.31 (30.20)	0.25
	**N (%)**	**N (%)**	
Sex ^2^			0.81
Male	22 (57.90)	221 (61.39)	
Female	16 (42.10)	139 (38.61)	
Race ^2^			0.80
White	23 (60.53)	237 (65.83)	
Black/African American	8 (21.05)	68 (18.89)	
Other	7 (18.42)	55 (15.28)	
Ethnicity ^3^			0.77
Non-Hispanic/Non-Latino	19 (50)	164 (45.56)	
Hispanic/Latino	16 (42.11)	171 (47.50)	
Unreported/Refused	3 (7.89)	25 (6.94)	
Primary Coverage ^3^			0.04 *
MAP/SFS	25 (65.79)	180 (50)	
Medicaid	1 (2.63)	49 (13.61)	
Medicare	11 (28.95)	89 (24.72)	
Other	1 (2.63)	42 (11.67)	
Statin Intensity ^2^			0.27
Low/Moderate ^4^	6 (15.79)	85 (23.61)	
High	32 (84.21)	275 (76.39)	
High-Risk SDOH ^2^			0.78
0	13 (34.21)	141 (39.17)	
1–2	13 (34.21)	114 (31.67)	
3–5	8 (21.05)	80 (22.22)	
6+	4 (10.53)	25 (6.94)	
Clinical Pharmacist Visit in 2023 ^2^			
No	23 (60.53)	275 (76.39)	0.05
Yes	15 (39.47)	85 (23.61)	
Number of ASCVD Indications ^2^			<0.01 *
1	8 (21.05)	204 (56.67)	
2+	30 (78.95)	156 (43.33)	

^1^ Wilcoxon Rank-Sum Test. ^2^ Chi-Square Test ^3^ Fisher Exact Test. ^4^ Categories combined due to low sample size. * Significant *p*-value.

**Table 3:** Variables by LDL-C Levels.

Variable	LDL-C < 70 mg/dL (*n* = 143)	LDL-C ≥ 70 mg/dL (*n* = 255)	*p*-Value
	**Mean ± SD (Median)**	**Mean ± SD (Median)**	
Age ^1^	59.80 ± 9.48 (61)	59 ± 7.90 (60)	0.10
BMI ^1^	30.30 ± 6.79 (29.40)	32.20 ± 7.50 (31.20)	0.01 *
	**N (%)**	**N (%)**	
Sex ^2^			1.00
Male	87 (60.84)	156 (61.18)	
Female	56 (39.16)	99 (38.82)	
Race ^2^			0.27
White	93 (65.04)	167 (65.49)	
Black/African American	23 (16.08)	53 (20.78)	
Other	27 (18.88)	35 (13.73)	
Ethnicity ^2^			0.01 *
Non-Hispanic/Non-Latino	51 (35.66)	132 (51.77)	
Hispanic/Latino	79 (55.25)	108 (42.35)	
Unreported/Refused	13 (9.09)	15 (5.88)	
Primary Coverage ^2^			0.70
MAP	73 (51.05)	132 (51.77)	
Medicaid	17 (11.89)	33 (12.94)	
Medicare	40 (27.97)	60 (23.53)	
Other	13 (9.09)	30 (11.76)	
Statin Intensity ^2^			0.86
Low/Moderate ^3^	32 (22.38)	59 (23.14)	
High	111 (77.62)	196 (76.86)	
High-Risk SDOH ^2^			0.73
0	59 (41.26)	95 (37.26)	
1–2	45 (31.47)	82 (32.16)	
3–5	31 (21.68)	57 (22.35)	
6+	8 (5.59)	21 (8.23)	
Clinical Pharmacist Visit in 2023 ^2^			
No	105 (73.43)	193 (75.69)	0.71
Yes	38 (26.57)	62 (24.31)	
Number of ASCVD Indications ^2^			0.89
1	75 (52.45)	137 (53.73)	
2+	68 (47.55)	118 (46.27)	
Use of Ezetimibe ^2^			0.26
Yes	10 (6.99)	28 (10.98)	
No	133 (93.01)	227 (89.02)	

^1^ Wilcoxon Rank-Sum Test. ^2^ Chi-Square Test. ^3^ Categories combined due to low sample size. * Significant *p*-value.

## Data Availability

The data presented in this study are available on request from the corresponding author due to privacy concerns.

## Abbreviations

The following abbreviations are used in this manuscript:

ASCVD	Atherosclerotic Cardiovascular Disease
BMI	Body Mass Index
CVD	Cardiovascular Disease
EHR	Electronic Health Record
FQHC	Federally qualified health center
LDL-C	Low-Density Lipoprotein Cholesterol
MAP	Medical Access Program
PAP	Patient Assistance Program
PCSK9i	Proprotein Convertase Subtilisin/Kexin Type 9 (PCSK9) Inhibitor
SD	Standard Deviation
SDOH	Social Drivers (or Determinants) of Health
